# Service Delivery Redesign for Noncommunicable Disease Management: Assessment of Needs and Solutions Through a Co-Creation Process in Argentina

**DOI:** 10.9745/GHSP-D-24-00208

**Published:** 2024-12-20

**Authors:** Agustina Mazzoni, Javier Roberti, Marina Guglielmino, Ana María Nadal, Yanina Mazzaresi, Andrea Falaschi, Patricia J. García, Laura Espinoza-Pajuelo, Jesús Medina-Ranilla, Hannah H. Leslie, Juan Manuel Gómez Portillo, María Gabriela Masier, Ezequiel García-Elorrio

**Affiliations:** a Institute for Clinical Effectiveness and Health Policy, Buenos Aires, Argentina.; b Ministry of Health of Mendoza Province, Mendoza, Argentina.; c Universidad Peruana Cayetano Heredia, Lima, Peru.; d Division of Prevention Science, University of California, San Francisco, San Francisco, CA, USA.; e Obra Social de Empleados Públicos de Mendoza, Mendoza, Argentina.

## Abstract

**Introduction:**

In Argentina, the implementation of a national strategy to reduce the prevalence of noncommunicable diseases (NCDs) has been hampered by challenges at the provincial level. We aimed to design a new model of care for NCDs at the primary care level by conducting a multimodal system assessment and co-design of potential solutions in the province of Mendoza.

**Methods:**

We carried out a mixed-methods study with 7 components: evaluation of patterns of care, patient focus groups, cross-sectional standardized population-based phone survey, an electronic cohort follow-up of patients with type 2 diabetes, in-depth interviews with stakeholders, a knowledge test for health care providers on chronic condition management, and a Delphi consensus to provide recommendations from stakeholders.

**Results:**

Focus group and in-depth interviews revealed access to primary health care for NCDs was associated with problems with long waiting times and time-consuming procedures for referral to laboratory tests, hospital care, and provision of medication. Mental health care services were particularly limited. Survey respondents (N=1,190) were predominantly covered through public (41%) or social security sectors (54%); 41% fell in the lowest income group. Contact with the health system was high (5.7 annual visits), but 19.7% reported unmet health care needs. Public sector providers perceived they provided high-quality care despite insufficient material and human resources. Within the social security sector, the main challenge was insufficient staff, particularly affecting mental health care. Health care providers showed a higher percentage of correct answers to depression-related questions, but worse results were seen in hypertension and diabetes care. Actions supported by evidence and expert agreement were identified for implementation to guide future system changes.

**Conclusion:**

Our research highlights the potential for Argentina’s primary care system to initiate transformative, system-level changes aimed at improving health outcomes. We propose an innovative methodological assessment and co-design for improving primary care.

## Abstract (precis)

Our research highlights the potential for Argentina’s primary care system to initiate transformative, system-level changes to improve health outcomes. We propose an innovative methodological assessment and co-design for improving primary care.

## Teaser

**Key Findings:**

- In conducting a comprehensive assessment of primary health care in Mendoza, Argentina, with a focus on noncommunicable diseases, we aimed to analyze patterns of health service use and disease management, user preferences for care and their perspectives on health system performance, and health care providers’ perspectives on health system performance.
- The results revealed barriers to patients accessing services exacerbated by financial constraints, as well as significant gaps in patient experiences, system competence, and trust in the public health system.
- One in 5 respondents to a population-based survey reported unmet health needs, providing valuable insights into users’ perspectives on health system performance. Limitations in access to mental health care were evident, reflecting wider systemic issues.
- A consensus process produced actionable recommendations for policymakers, providing suggestions to guide future health system changes and research agendas based on evidence-based practices.

**Key Implication:**

- Using inclusive, equity-focused methods to assess health system performance ensures that the perspectives of underserved populations are addressed in the co-design process and highlights disparities in access to health care services.

## INTRODUCTION

Primary health care (PHC) plays a critical role in saving lives and optimizing resources, contributing to more resilient and effective health systems that benefit all. The COVID-19 pandemic exacerbated existing vulnerabilities, with serious health and economic consequences. However, it also provided a unique opportunity for transformative change in health systems, particularly in the redesign of PHC to meet emerging needs and fill existing gaps in care, especially for chronic conditions.^1^

Adapting PHC to meet the needs of the evolving health care landscape will require a rethinking of governance and accountability within PHC systems. Governance and accountability structures play a central role in influencing the dynamics between patients, providers, and stakeholders, as well as in mediating the different interests and power dynamics among the latter. Therefore, it is essential to understand the motivations of all relevant actors or those who should be involved when undertaking a redesign process at the PHC level.^2^ As noted by The Lancet Global Health Commission on High Quality Health Systems, the foundations of high-quality health systems include the population and its health needs and expectations, health sector governance and cross-sector partnerships, platforms for care delivery, workforce numbers and skills, and tools and resources, such as medicines and data.^2^ The Commission led to a global effort called Quality Evidence for Health Systems Transformation (QuEST), launched by the Harvard T.H. Chan School of Public Health, to strengthen health systems and improve their quality of care based on research evidence and the creation of QuEST Latin America and the Caribbean (QuEST LAC), an innovation hub focused on LAC to provide locally adapted and usable inputs. This cluster is currently led by the Institute for Clinical Effectiveness and Health Policy in Argentina and Cayetano Heredia University in Peru.^3^

Service delivery redesign (SDR) is an innovative, system-level framework for improving care, defined as the reorganization and strengthening of existing services and care pathways to maximize the quality of care and optimize health outcomes. It can also be defined as the deliberate reorganization of a health system to improve equity, quality, and outcomes. SDR involves the following stages: feasibility assessment (including gap analysis), co-design, implementation, and evaluation.^4^

Argentina is an upper-middle-income country in South America with a population of 44 million people, 92% of whom live in urban areas. In a country that has almost completed its demographic, epidemiological, and nutritional transition,^5^ noncommunicable diseases (NCDs) account for more than 78% of the burden of disease,^6^ while cardiovascular disease accounts for about one-third of NCD-related deaths. Argentina has a highly developed health system, especially by the standards of low- and middle-income countries in the region, and its health system performs well on several key indicators. However, health outcomes fall short of the country’s potential given that it is among the leaders in the region in terms of per capita health expenditure and human development. In short, as in many other countries in Latin America, Argentina’s major health problems today are related to both equity and efficiency.^5^

The health care system in Argentina comprises 3 sectors.

- Public sector: Financed by the Ministry of Health (MOH), its main beneficiaries are persons without health insurance, usually from lower socioeconomic groups. At least 36% of Argentina’s population is covered only by the public health system.
- Social security sector: Grounded in the social insurance principle, which requires all employers and employees to make payments to a trust fund, this sector provides services for a variety of institutions, which vary greatly depending on the type of employment and medical insurance provided. The social insurance sector provides health coverage to 60% of the population.^6^
- Private sector: Provides services to individuals of high socioeconomic status who may have different types of prepaid health insurance packages.^7^

In 2009, the National MOH established the National Strategy for the Prevention and Control of NCDs through Ministerial Resolution 1083/09, based on the Model of Approach to People with Chronic Diseases (MAPEC in Spanish). This strategy aims to reduce the prevalence of risk factors for NCD mortality and improve access and quality of care. However, due to the organizational structure of the Argentine health system, which is federal and decentralized, the implementation of a national strategy for NCDs depends largely on the political will of the provinces. While the National MOH provides strategic direction and develops clinical practice guidelines, their adaptation and implementation at the local level are primarily the responsibility of provincial authorities.

Mendoza, a province in Argentina, exemplifies these challenges. Despite the widespread availability of PHC providers, hospitals at secondary level or higher continue to treat a significant number of patients with chronic conditions that could be managed at the primary care level. This may be attributed to factors such as a lack of trust in providers from the users’ perspective, perceived limitations in their skills to manage common chronic conditions, long waiting times, and problems with scheduling, among others.^8^ From a supply-side perspective, PHC providers may lack confidence in their ability to diagnose and treat chronic conditions, lack appropriate medication and diagnostic tests, or have poor customer service.^8^

Despite the widespread availability of PHC providers in Mendoza, hospitals at secondary level or higher continue to treat a significant number of patients with chronic conditions that could be managed at the primary care level.

In this context, we aimed to design a new model of care following the first 2 steps of the SDR process: multimodal system assessment and co-design of potential solutions for the PHC level focused on persons with NCDs in the province of Mendoza, Argentina.

## METHODS

### Methodological Approach and Objectives

This study was carried out using the QuEST network’s tools and methodologies.^4^ Our approach highlighted the key stages of model co-creation, underscoring relevant sources of information at each stage. Our focus was on the design phase, which involved rethinking governance and accountability structures within PHC systems. Our approach recognized Argentina’s unique health care landscape and emphasized the need for a comprehensive approach to serving diverse health care user groups.

The objectives for the system assessment and co-design of potential solutions were to: (1) describe patterns of health service use and disease management for people with 3 chronic conditions (hypertension, diabetes, and depression), selected based on prevalence, and analyze the factors influencing inappropriate use of primary care in these patients; (2) generate empirical evidence on people’s (users’ and non-users’) preferences for their health care and views on health system performance to inform the redesign of primary care for the general population, with a particular focus on people with NCDs; (3) assess the perspectives of health care providers and decision-makers on the feasibility of implementing the reforms proposed by the community; and (4) propose a locally feasible primary care model based on the results of the previous objectives through a co-creation process with all stakeholders to be implemented and evaluated in future studies.

To achieve these objectives, we conducted a mixed-methods study with 7 different components. The Table summarizes these components, objectives, and corresponding methods of the coproduced care model design process, which will be further explained.

**TABLE.** Components, Objectives, and Methods of Coproduced Care Model Service Delivery Redesign Process, Argentina

**Phases**	**Component**	**Objective**	**Methods**
**Feasibility assessment**	Evaluation of patterns of care	To assess health system utilization patterns and the degree of control for diabetes, hypertension, and depression.	Secondary analysis of databases in 3 steps: Identification of patients with ≥1 of 3 conditions through clinical records Pattern of health system utilization Analysis of level of disease control using hemoglobin A1C in diabetes and blood pressure measurements in hypertension. Continuity of care was explored for a subgroup of patients receiving antidepressant medication.
	Focus groups with persons with hypertension, diabetes, and depression	To evaluate the burden of treatment experienced by patients with hypertension, diabetes, and depression.	Qualitative exploratory study; 10 focus group sessions at primary health care centers of both subsectors.
	In-depth interviews with stakeholders	To assess program activities and objectives, services, characteristics of the population served, interactions with other levels of care, and challenges and facilitators for care.	Qualitative study; 19 in-depth interviews with health care providers and policymakers from the 2 subsystems.
	Survey on people's views on health system performance. (People's Voice Survey)	To measure how users interact with and perceive the health system.	A total of 1,190 adults in Mendoza were surveyed via telephone through a known list sampling approach, using a quota sampling based on gender, age, department, and insurance coverage.
	Electronic cohort of diabetic patients	To evaluate the performance of the health system in managing type 2 diabetes.	252 people from both subsectors were followed up via mobile phone for 6 months and administered a specially designed survey (e-cohort).
	Knowledge test to health care providers	To explore weaknesses in providers’ skills to manage noncommunicable diseases.	A test was embedded in a training course offered by the Ministry of Health to providers in both subsectors. Questions were based on current provincial and national guidelines.
**Co-design**	Consensus process to develop recommendations	To establish a list of evidence-based recommendations to improve the primary care network.	A modified Delphi RAND UCLA method was used to build consensus.

### Theoretical Framework

Health care is not a commodity produced by the health care industry but a service coproduced by health care professionals in collaboration with each other and with patients seeking help to restore or maintain their health and the health of their families. This coproduced partnership is influenced or hindered by various factors operating at both the health system and community levels. Coulter and colleagues presented a House of Care model, which represents a collaborative approach to the management of chronic health conditions.^9^ This model emphasized personalized care planning supported by responsive policy and governance; organizational processes and workflows; and the skills, dispositions, and behaviors of health professionals and patients. Wagner’s chronic care model emphasized the importance of engaged patients working with prepared professionals to achieve functional and clinical outcomes, recognizing the critical support provided by community and health system resources.^10^ Based on these models, a coproduced model of health care was proposed in which patients and professionals act as participants within a societal health care system. The health care system and the social forces of the wider community play a role in supporting and constraining partnerships between patients and health professionals. Both patients and professionals have the ability to shape the system as participants. Patients and the public interact with individuals and organizations outside the health care system to influence health and health care outcomes.^11^ Coproduced health services then contribute to overall health, which is influenced by different social factors and sources of care. Coproduction blurs the roles of patients and professionals and extends beyond the health system into the wider community. The model recognizes different levels of co-creative relationships, from basic civil discourse and respectful interaction to joint planning and implementation. Each level requires specific knowledge, skills, attitudes, and behaviors.^11^

### Setting

Mendoza, a province with a population of 2 million people and an area of 148,000 km^2^, has 25 hospitals and 342 PHC centers, organized into 5 health regions covering 18 departments. PHC centers and hospitals are under provincial jurisdiction. Over the past 8 years, the Institute for Clinical Effectiveness and health Policy has worked closely with health policymakers in Mendoza, Argentina, researching and sharing results with the MOH on cancer screening policies and developing interventions for screening and management of cardiovascular patients at the primary care level. Chronic disease management programs have been implemented at the primary level in the public and social security subsectors.

### Assessment

For this phase, we applied the following components of our analysis, some of them including patients as research subjects and some others considering health care providers and other stakeholders as its population. The assessment had the following components.

#### Evaluation of Patterns of Care

We conducted a secondary analysis of the public and social security subsectors databases to assess patterns of health system use and levels of disease control for specific conditions in 2019: diabetes, hypertension, and depression. In this component of the study, we aimed to analyze the subgroup of individuals who successfully engage with the health care system and assess their utilization patterns. In contrast, other components, such as the People’s Voice Survey, included non-users, allowing us to capture insights from both users and non-users of the system. These databases were provided by the MOH for the public sector and by the authorities of the provincial social security for this sector. The databases are not public, and we have access to deidentified databases.

To identify patients diagnosed with 1 or more of these conditions, we used records of specific registered medications. In public sector databases, patients were also identified by diagnosis at consultation in electronic clinical records.

Once identified, patterns of health system use were analyzed by recording the level of care at which they attended and the number of visits per year. In addition, the level of disease control was analyzed using hemoglobin A1C (HbA1C) blood test in diabetic patients and blood pressure (BP) measurements in hypertensive patients. However, providers did not use depression scales in the analysis. Continuity of care was examined and defined as the receipt of antidepressant medication for at least 6 months per year. Finally, patterns of provider visits and type of care received were analyzed.

#### Qualitative Study on Primary Health Care Users

This qualitative inquiry aimed to assess the burden of treatment^12^ experienced by patients with hypertension, diabetes, and depression while navigating the PHC system.

A qualitative inquiry aimed to assess the burden of treatment experienced by patients with hypertension, diabetes, and depression while navigating the PHC system.

Ten focus group sessions (FGS) were conducted in PHC centers of public and social security subsectors. A purposive sampling approach was used; inclusion criteria were patients older than age 18 years with a confirmed diagnosis of diabetes, hypertension, or depression who used PHC services in Mendoza. The sessions were conducted in August and October 2022 in the capital, small towns, and rural areas of Mendoza. Focus group sessions were conducted separately for women and men, without the presence of any staff member, with an average duration of 1 hour and an average of ten participants per session.

A flexible interview guide based on the main constructs of the Burden of Treatment theory by May et al. and the conceptual framework of access to health care by Levesque et al.^12^^,^^13^ facilitated the discussions, which covered participants’ experiences of diagnosis, access to services, health care preferences, management of conditions, and barriers to care. Sessions were audio-recorded with participants’ informed consent, and pseudonyms were assigned. All transcripts were uploaded and managed using Atlas.ti version 8.1.3 (Scientific Software Development GmbH, Germany). Data were analyzed using an abductive approach, which combines elements of inductive and deductive reasoning and allows researchers to move iteratively between the empirical data and existing theoretical frameworks.^14^ A collaborative coding process was used. JR developed a preliminary coding framework based on the theory, then identified themes from the data, and shared it with other researchers. Throughout the analysis, the team engaged in discussions to refine the coding scheme, ensuring that it accurately reflected the theoretical underpinnings and the participants’ lived experiences.

#### In-Depth Interviews With Stakeholders

We conducted 19 in-depth interviews with health care providers and policymakers from the 2 subsystems. A purposive sampling approach was used and included directors of chronic disease programs, directors of primary care centers, primary care physicians and psychologists, and secondary care specialists. In the interviews, we assessed the following areas: program activities and objectives, services available, characteristics of the population served, interactions with other levels of care, difficulties, challenges, and facilitators for patient care. All interviews formed a coherent corpus. Interviews were audio-recorded and transcribed verbatim; then, all transcripts were uploaded to the qualitative software program Atlas.ti v8.1.3. The coding process involved a combination of inductive coding, where themes emerged from the data, and deductive coding, based on preexisting theoretical constructs.^14^ This dual approach ensured a comprehensive analysis, capturing predefined categories and unexpected insights that contributed to a deeper understanding of the practices to manage conditions. A collaborative coding process was employed to ensure rigor and reflexivity in the analysis.

#### Survey on People’s Views on Health System Performance

The People’s Voice Survey, developed by the QuEST network,^15^ is a rapid, low-cost, population-based telephone survey designed to promote health systems’ accountability, track the impact of reforms and policies over time, promote benchmarking across countries and subnational regions, and inform action toward more effective and person-centered health systems. We conducted cognitive interviews and pilot testing before administering the survey in Mendoza. In September 2022, data were collected from a sample of 1,190 adults in Mendoza. The survey aimed to obtain population sentiment about the performance of the health system by estimating population proportions agreeing with a range of statements. A survey of 1,000 individuals selected at random will produce an estimate that is within a 3% margin of error of the population proportion 95% of the time. This is the case when the prevalence is 50%; smaller numbers are needed when the prevalence is higher or lower. Thus, we used a minimum sample of 1,000 in Argentina.^15^ The survey was conducted by IPSOS Argentina using telephone interviews and a known list sample, with quota sampling based on gender, age, department, and insurance coverage. No post-stratification weights were needed. Before data collection, interviewers received formal training on the survey to ensure that they understood the study objectives, purpose of each survey question, potential barriers to obtaining responses, and potential respondent problems and how to address them. The training sessions were conducted by QuEST researchers and IPSOS trainers.

#### Electronic Cohort of Patients With Diabetes

This was a longitudinal, prospective, real-time survey designed to evaluate the performance of the health care system, assess patients’ experiences of managing their disease, and identify gaps along the continuum of care for people with type 2 diabetes. This component was started in March 2023 and finalized in March 2024. The diabetes electronic cohort’s (e-cohort) 4 goals were to (1) evaluate the feasibility of using electronic cohorts in chronic disease populations as a flexible measurement tool to assess health system performance using cell phones, (2) measure the competence of the health system to care for patients with chronic diseases, (3) describe the user experience and processes of care in the health system, and (4) identify weaknesses in the delivery of effective care for good health outcomes.

We recruited 252 people from the public and social security sectors and followed them virtually (using mobile technology) for 6 months. Cognitive interviews were conducted before recruitment began to test and adjust the instrument. The e-cohort included 3 different phone surveys: a baseline survey, a monthly follow-up survey, and an endline survey performed at month 6. The interviewers were staff from the MOH in the public sector and staff from the call center in the social security sector, specially trained to conduct the survey and use RedCap for data entry.

#### Knowledge Test for Health Care Providers

This assessment was embedded in a training course offered by the MOH to public and social security health providers in Mendoza. The skills assessment questions were based on current provincial and national guidelines. We aimed to determine whether there were any weaknesses in providers’ ability to meet the needs of patients with chronic conditions. Participation was voluntary, and 99 of 300 invited health care providers completed the survey.

#### Consensus Process to Develop Recommendations

The purpose of the consensus process was to reach agreement on recommendations and generate a comprehensive list of feasible and culturally appropriate interventions specifically adapted to the Mendoza health system. The aim was to provide the province with evidence-based recommendations that would have a positive impact on the primary care network, particularly in chronic disease management.

The consensus process aimed to provide the province with evidence-based recommendations that would have a positive impact on the primary care network.

A modified RAND UCLA Delphi method was used to reach consensus.^16^ This method is widely used to reach consensus among groups of experts or stakeholders on a particular issue.^17^^,^^18^ The Delphi process is structured and iterative to gather opinions and ideas from a group of participants. This iterative process promotes convergence toward consensus, leading to a final set of recommendations or decisions. A local steering group composed of researchers, members of the MOH, and experts developed a list of potential interventions to improve the Mendoza provincial health system. The group reviewed evidence from multiple sources, including the People’s Voice Survey, an electronic cohort of diabetic patients, qualitative studies of user and provider experiences, and relevant evidence on primary care interventions. A total of 32 experts representing different levels and roles in the health care system, selected for their public health expertise, were invited to participate in a consensus process. Twenty-two experts participated in 2 rounds of online voting using a questionnaire hosted on Zoho. Each recommendation was rated on 5 criteria (priority, impact, resource requirements, acceptability, and feasibility) from the Grading of Recommendations Assessment, Development and Evaluation Evidence to Decision framework endorsed by the World Health Organization. Experts rated each criterion on a 9-point Likert scale. The RAND/UCLA Appropriateness Method was used to determine expert agreement on each criterion of each recommendation. Recommendations were then classified as adequate (median score of 7–9 with no disagreement), uncertain (median score of 4–6 or with disagreement), or inappropriate (median score of 1–3 with no disagreement) based on the scores for the criteria and the presence of agreement on these scores. Experts were given 10 days to complete each round, with reminders for non-responders. In cases of disagreement, recommendations were reassessed in subsequent rounds. The final consensus meeting was held in Mendoza on July 6, 2023, with 20 experts attending to discuss recommendations where there was disagreement. After thorough discussion, a final list of recommendations was compiled and sent to the expert group for feedback.

### Ethical Approval

The study, including all the described components, was approved by the Ethics Committee of Mendoza Province. Informed consent forms were signed by the participants of the focus group sessions and the in-depth interviews with stakeholders. Both for the People’s Voice Survey and the e-cohort, verbal informed consent was requested. The knowledge test for health care providers was done as a pre-test of a course conducted by the MOH directed to primary care providers, and they signed an informed consent form.

## RESULTS

We describe the key findings of each component and how they led to the development of solutions by local stakeholders. Detailed information on the results of each component can be found in the Supplement.

### Patterns of Care

We identified 25,124 people with diabetes, 56,019 people with hypertension, and 20,399 people with depression in the available databases from the public system of 2019. Of these, 67% of people with diabetes used the PHC level to receive care, 66% of people with hypertension, and 59% of people with depression. Within the social security sector, in 2019, we identified 22,333 people with diabetes, 56,460 with hypertension, and 27,923 with depression. The utilization of PHC within the social security sector exceeded that of the public sector across all 3 groups: 90% of people with diabetes, 89% of individuals with hypertension, and 86% of those with depression. Concerning disease control, only 15% of the patients who were identified as having diabetes in the public sector had a record of having undergone HbA1C measurement compared to 33.6% in the social security sector. Of these patients, 38% and 61% had an HbA1C value of less than 7% in the public and social security sectors, respectively. If 8% is the cutoff point for HbA1C, these proportions are higher in both sectors (54% and 77%, respectively).

Among patients with hypertension, 23% had 1 or more BP records in the public sector and 39% in the social security sector. Of these, 63% and 66% were under control (systolic BP<140 mmHg and diastolic BP<90 mmHg) in the public and social security sectors, respectively. Regarding persons with depression, assessing disease control was not possible, primarily because health care providers in the province did not use depression scales. Continuity of care was measured in the subgroup of patients who received at least 6 packs of antidepressant medication in the year. In this subgroup of patients, the median number of visits for patients in the public sector (N=1,849) was 15 and in the social security (N=11,264) was 9. In the public sector, 5% of the visits were to a mental health care provider, and only 6% in the social security sector, and 40% of the visits were to a general or family physician.

### Burden of Treatment Experienced by Patients

The focus group sessions revealed the impact of the burden of treatment, including users’ efforts in securing care and accessing medication; the physical and emotional impact of the burden of treatment; factors influencing it, like system saturation and financial burden; and strategies to enhance capacity through networking, resource mobilization, and careful planning, among others. Access to PHC centers was challenging for patients, including long waits for appointments booked through phone apps or special numbers. Obtaining medicines, especially in the public system, required repeated visits due to inconsistent availability. Laboratory tests and hospital care required time-consuming processes. The burden of treatment disrupted daily life and created feelings of helplessness. Financial constraints exacerbated these challenges, leading some patients to seek alternative subsystems or rely on support networks. Strategies, such as using emergency services and careful planning, were used to navigate the system. However, limitations in access to mental health care persisted, contributing to the complex burden that patients faced. Despite the use of primary care services, disease control measures varied, highlighting systemic issues that impacted effective management. Patients’ reliance on support networks and proactive planning emerged as coping mechanisms in the face of health system deficiencies.

Focus group session participants discussed the impact of burden of treatment, the physical and emotional impact of the burden of treatment, and factors influencing it.

### Stakeholders’ Perspectives on Health System Performance

During in-depth interviews, stakeholders within the public health care system reported that they perceived they were delivering high-quality care, yet they acknowledged deficiencies in both material and human resources. They emphasized insufficient staff as the primary obstacle and suggested solutions, such as extending work hours and reallocating responsibilities. According to interviewees, the scarcity of human resources hampered health promotion and disease prevention efforts. In the area of mental health, the shortage of psychiatrists significantly impacted care provision. Conversely, within the social security sector, the predominant challenge was the lack of personnel to address the substantial patient demand, particularly evident in mental health services due to a scarcity of psychiatrists and limitations in referrals to public hospitals. While the introduction of group therapies might alleviate the workload, users’ preferences for individual consultations further complicate the scenario.

### Users’ Perspectives on Health System Performance

A total of 1,190 users and non-users were surveyed during September, October, and November 2022. The survey encompassed a population with a median age of 49 years, predominantly female (61%), and mainly urban, though rural areas were also included. Public sector coverage was held by over 40%, provincial social security (Obra Social de Empleados Públicos de Mendoza [OSEP]) by 18%, other social security coverage by 26%, and private coverage by 8.5%. Around 37% reported chronic illnesses, notably rising to 60% among those aged 60 years and older. Overall, contact with the health system was high (5.7 annual visits), and only 19.7% reported unmet health care needs. About 4.6% reported poor health, with a higher proportion among those aged 50–60 years and older than 60 years. Slightly over half displayed low health activation levels, particularly prevalent among lower education and income groups. Approximately 5% had low expectations regarding health care quality.

Overall, contact with the health system was high, and only 19.7% reported unmet health care needs.

More than 80% of respondents reported having a frequent health care source corresponding to their coverage. For public facilities, proximity was the main reason, while OSEP facilities were chosen due to insurance coverage. In the private sector, care quality drove choice. Approximately 12.8% had teleconsultations in the past year, with lower-income, male, and rural respondents less likely to benefit. Nearly 87% had in-person consultations in the prior year, half for prevention and others for acute/chronic issues. Twelve percent reported feeling discriminated against, and over 10% noted errors in care. Compliance with annual preventive actions varied from 45% to 70%. Public and OSEP facilities had longer wait times than in the private sector. Over 50% rated their care as very good or excellent; this percentage was higher in private facilities. Overall, there was a positive perception of health subsystems, slightly lower among non-users. However, confidence in health security and government responsiveness was low. Thirty percent believed the system was improving, while 70% felt it stagnated or worsened. Only 9% suggested a complete overhaul, but 70% felt major changes were necessary for improvement. These insights should inform health care reforms, focusing on areas with lower ratings. The comparative results of the People’s Voice Survey were published elsewhere.^15^^,^^19^^–^^21^

### Health System Performance Managing Type 2 Diabetes

In the diabetes e-cohort, a total of 252 patients were surveyed, with 172 individuals from the public sector and 80 from the social security sector. The mean age was 58 years (standard deviation: 18.3); 61.3% were female; .41.1% had completed at least secondary education, 19.1% belonged to the lowest household income level, and 53% were employed. Regarding the continuity of care, during the follow-up months, 44.4% (57.5% public sector, 16.3% social security) responded to 2 or more telephone surveys. Among them, 29.1% reported on a scale of 1–100 that their health status was equal to or greater than 80, and the quality of care received was rated as excellent or very good by 63.5%. Most survey participants required 15 minutes to reach the health care facility they visited. The waiting time and the duration of the visit with the health care provider were both approximately 30 minutes.

### Providers’ Skills Managing Noncommunicable Diseases

On the skills assessment, health care providers showed a higher percentage of correct responses on questions related to depression: 99% knew the cardinal symptoms, 92% were able to describe those symptoms, 99% were aware of the differential diagnosis of depression, and almost 70% knew about the treatment of depression. Regarding hypertension, almost 60% were aware of the best type of sphygmomanometer, 62% knew the target range for BP control, and 48% knew the optimal frequency for BP control. Finally, for diabetes, providers showed more knowledge about diabetes diagnosis and lifestyle changes (83% and 96%, respectively) and lower percentages of correct responses in 3 different treatment cases presented as examples (51%, 45%, and 32%).

### Process to Develop Evidence-Based Recommendations

The steering group and a small group of experts evaluated the findings from different components to develop a list of potential interventions to improve the provincial health system. Twenty-two professionals participated in the first 2 rounds of voting to evaluate the recommendations according to 5 criteria proposed by GRADE from the Evidence to Decision framework. The final round of voting, attended by 20 participants, was held in Mendoza to discuss the recommendations that showed disagreements in the scoring of the evaluation criteria and to reach a consensus whenever possible.

The findings of the consensus showed numerous actions that are supported by evidence and expert agreement for implementation, which will guide future changes in the system or be part of the research agenda (Box).

BOX Consensus Evidence-Based Recommendations to Improve the Provincial Health System in Mendoza, Argentina

The initiatives with the highest consensus scores were:

1. Implement the digital transformation law.
2. Promote mechanisms for user participation in the design and provision of health care in the health system.
3. Develop skills in users to make them active participants in health care.
4. Use information and communication technologies to provide information on specific conditions, clinical reminders, screenings, and appointment cancellations.
5. Develop coproduced definitions of quality care involving both users and health care personnel at the primary care level.
6. Implement an incident reporting system in primary care centers.
7. Implement performance metrics to assess professional practice.

## DISCUSSION

In our collaboration with the MOH of Mendoza, we conducted a comprehensive assessment of primary care with a focus on NCDs. This assessment was carried out using the QuEST network’s tools and methodologies, including a population-based survey, a cohort study, and health system data analysis. The results of the assessment highlighted deficiencies in patient experience in navigating the system, the system’s competence, and people’s trust in the health system. To address these shortcomings, we used the Delphi process to engage experts in formulating recommendations to improve quality, accessibility, and patient-centered care. These recommendations are a critical first step in transforming the health system in Mendoza.

The results of the assessment highlighted deficiencies in patient experience, the system’s competence, and people’s trust in the public health system.

The results revealed barriers to accessing PHC services, such as long waiting times, difficulties in obtaining medicines, and time-consuming procedures for laboratory tests and hospital care. These challenges have a significant impact on patients’ daily lives and are exacerbated by financial constraints. As a result, patients often turn to alternative sources of care and social support networks because of the strain on the health care system. The People’s Voice Survey revealed demographic nuances, but respondents perceived that public and social insurance coverage was adequate and that health care use was high. Stakeholder interviews highlighted the inadequacy of material and human resources within the public health system, including staff shortages, and suggested possible solutions. Finally, the consensus process produced actionable recommendations for policymakers, providing evidence-based suggestions to guide future health system changes and research agendas.

To our knowledge, there are no similar published studies evaluating the health system in Argentina. Previous studies have mainly focused on access to the health system and equity and were published more than 10 years ago.^22^^,^^23^ There is only 1 published study analyzing the relationship between social determinants, gender, with inequities of access in frequent users of the public health system in 1 province of Argentina using a cross-sectional methodology in 2018.^24^

The current health system in Argentina faces challenges in effectively addressing NCDs. Despite the National Strategy for the Prevention and Control of NCDs, decentralized implementation at the provincial level depends on local political will and resources.^25^^,^^26^ Given the challenges identified, there is an urgent need to implement evidence-based interventions to improve primary health care, particularly for the management of chronic diseases. The urgency is heightened by the post-pandemic context and the impact of economic challenges, making it imperative to rapidly redesign the first level of care for the general population.^27^^,^^28^

The methodology employed followed a theoretical framework that guides system redesign in health care delivery.^29^ This theoretical framework proposes that health care is a service that is co-created by patients and health care professionals and influenced by various factors at both the systemic and community levels. The model emphasizes personalized care planning and engages patients in collaboration with trained professionals to achieve favorable outcomes. Challenges in the primary care system highlight the need for collaboration to address systemic inefficiencies. The findings provide critical insights into user and non-user perspectives on health system performance and inform evidence-based recommendations that are essential to guide the ongoing process of health system transformation in Mendoza. Our study takes a coproduction approach, recognizing the distinctive nature of health care services as fundamentally coproduced entities. This paradigm encourages the exploration of innovative approaches to health professional education, patient socialization, organizational structures, and performance metrics, offering a valuable perspective for transforming primary care.^11^

We plan to conduct a study to optimize and evaluate the effectiveness of a co-designed primary care model. The primary objective of that study would be to improve diabetes management and patient experience. Secondary objectives would include evaluating the feasibility and acceptability of the model; assessing its impact on health service utilization, system performance, user confidence, and cost per visit; and investigating its potential impact on inequalities related to socio-demographic factors. To achieve these objectives, we will conduct a cluster randomized controlled trial in PHC centers.

### Strengths and Limitations

There are potential limitations to the assessment process. Sampling bias in both the qualitative and quantitative components may limit the generalizability of the results, as certain demographic groups may be underrepresented. The People’s Voice Survey, which was conducted by telephone, carries the risk of selection bias, social desirability in responses, and recall issues. The Delphi consensus method is not free from bias in participant selection and challenges in reaching consensus, which may affect the validity of recommendations. In addition, the temporal limitations of the cohort study may not capture evolving patterns of care. As a strength of our study, the inclusion of both qualitative and quantitative components enriches the depth of understanding. The inclusion of a qualitative study adds richness that may provide valuable insights into nuanced aspects of the health care experience. Finally, the commitment to rigorous methodology, ongoing data collection, and cultural sensitivity demonstrates a proactive approach to mitigate potential bias and enhance the robustness of the findings.

## CONCLUSION

Our research highlights the potential for Argentina’s primary care system to initiate transformative, system-level changes aimed at improving health outcomes for people with type 2 diabetes. Our approach, which proposes a novel methodological framework for improving primary care, emphasizes the key stages of model co-creation, particularly the design phase, which involves the redesign of governance and accountability structures within primary care systems. This framework not only aligns with the QuEST initiative and the Lancet Global Health Commission on High-Quality Health Systems but also foregrounds the ethical imperative of promoting equity in health care. By employing inclusive methods like the People’s Voice Survey and co-design, the approach ensures that the perspectives of underserved populations—those most affected by systemic inequities—are integral to the redesign process. This is an equity-focused approach, with an emphasis on addressing the specific barriers faced by different population groups, highlighting disparities in access to health care, and proposing interventions to improve care. This democratic, participatory methodology fosters inclusivity and accountability, producing policy recommendations that are deeply rooted in the real-world experiences of people and health care providers and reinforces the ethical foundation of justice and accessibility. As a pioneering step in health care transformation, this methodology contributes to the broader discourse on quality health systems but also offers a blueprint for creating socially just inclusive policies that translate directly into legislative action. By embedding equity into both the process and outcomes, our integrated approach ensures that future health system reforms address existing disparities and promote long-term structural change for vulnerable populations.

## Supplementary Material

GHSP-D-24-00208-Supplement.pdf

## Notes

**Peer Reviewed**

**First Published Online:** December 11, 2024.
