# Supplementary material for: Service Delivery Redesign for Noncommunicable Disease Management: Assessment of Needs and Solutions Through a Co-Creation Process in Argentina
Source: Glob Health Sci Pract. 2024 Dec 20;12(6):e2400208. doi: 10.9745/GHSP-D-24-00208 (PMC11666083; doi:10.9745/GHSP-D-24-00208)
Supplement: GHSP-D-24-00208-Supplement.pdf [file GHSP-D-24-00208-Supplement.pdf]

Supplement to: Mazzoni A, Roberti J, Guglielmino M, et al. Service delivery redesign for noncommunicable disease management: assessment of needs and solutions through a co-creation process in Argentina. *Glob Health Sci Pract*. 2024;12(6):e2400208. https://doi.org/10.9745/GHSP-D-24-00208

Supplement. Summary of results

Characteristics of the database of diabetic, hypertensive and depressed patients 2
Qualitative study on system users 3
In-depth interviews with stakeholders 6
People Voice Survey (PVS) 7
Electronic cohort of patients with diabetes 9
Knowledge test to healthcare providers 12
Consensus process to develop recommendations 12

Supplement to: Mazzoni A, Roberti J, Guglielmino M, et al. Service delivery redesign for noncommunicable disease management: assessment of needs and solutions through a co-creation process in Argentina. *Glob Health Sci Pract*. 2024;12(6):e2400208. https://doi.org/10.9745/GHSP-D-24-00208

Characteristics of the database of diabetic, hypertensive and patients with depression

Table 1. General characteristics of the database patients in both subsectors (public and social security -OSEP-). Year 2019.

	Diabetes		Hypertension		Depression	
	**Public (N=25,124)**	**OSEP (N=22,333)**	**Public (N=56,019)**	**OSEP (N=56,460)**	**Public (N=20,399)**	**OSEP (N=27,923)**
**Demographics**						
Sex female	15,308 (61%)	12,049 (54.0%)	34,035 (61%)	32,431 (57.4%)	13,490 (66%)	20,277 (72.6%)
Older than 45 years	17,339 (70%)	20,048 (89.7%)	38,287 (68.7%)	53,074 (94.1%)	11,335 (55%)	22,168 (79.4%)
**Disease control**	**Hb1Ac < 8%**		**Blood pressure systolic BP <140 and diastolic BP <90)**		**≥ 6 packs of antidepressant in 2019***	
Under control	2,171 (54%)	5,766 (76.9%)	10,112 (78%)	14,662 (66.1%)	1849 (47%)	11,264 (40.3%)
Uncontrolled	1,821 (46%)	1,735 (23.1%)	2,909 (22%)	7,518 (33.9%)	2078 (53%)	16,659 (59.7%)
Missing data	21,132 (84%)	14,831 (66.4%)	42,985 (76.7%)	34,280 (60.7%)	16,472 (80.7%)	0 (0%)
**Level of care**						
Primary level	16,775 (67%)	20,169 (90.3%)	36,846 (66%)	50,316 (89.1%)	12,083 (59%)	23,955 (85.8%)
Secondary level	11,103 (44%)	7,344 (32.9%)	25,270 (45%)	18,619 (33.0%)	11,877 (58%)	9,638 (34.5%)
Tertiary level	5,455 (22%)	7,146 (32.0%)	8,399 (15%)	17,908 (31.7%)	4,811 (24%)	9,006 (32.3%)
**Number of visits (Median; IQR)**	6 (3-11)	9 (4-15)	5 (2-9)	9 (4-15)	7 (3-14)	8 (3-15)
**Number of visits by specialty in the primary health care level**						
	N= 111,508	N= 72,801	N= 213,973	N= 174,560	N= 94,304	N= 36,351
General physician	64,437 (58%)	33,233 (46%)	130,149 (61%)	73,668 (42%)	43,871 (47%)	36,794 (42%)
Other specialties	47,071 (42%)	39,568 (54%)	83,824 (39%)	100,892 (58%)	50,433 (53%)	50,245 (58%)

*Regarding persons with depression, assessing disease control was not possible primarily because healthcare providers in the province did not use depression scales. Controlled disease was measured in the subgroup of patients who received at least 6 packs of antidepressant medication during the year.

Supplement to: Mazzoni A, Roberti J, Guglielmino M, et al. Service delivery redesign for noncommunicable disease management: assessment of needs and solutions through a co-creation process in Argentina. *Glob Health Sci Pract*. 2024;12(6):e2400208. https://doi.org/10.9745/GHSP-D-24-00208

Qualitative study on system users

This qualitative inquiry aimed to assess the burden of treatment experienced by patients with hypertension, diabetes and depression while navigating the primary healthcare system. Ten focus group sessions (FGS) were conducted in PHC centers of public and social security sub-sectors. A purposive sampling approach was used; inclusion criteria were patients over 18 years old with a confirmed diagnosis of diabetes, hypertension or depression who used PHC services in Mendoza. The FGS were conducted in August and October 2022 in the capital, small towns, and rural areas of Mendoza. *See table 2.*

Table 1. Characteristics of focus groups

Focus group	Location	Subsector	Number of participants	Sex
1	Town	Public	5	Men
2	Town	Public	15	Men
3	City	Social security	10	Men
4	City	Public	15	Women
5	City	Social security	5	Women
6	City	Public	10	Women
7	Town	Social security	7	Women
8	Town	Social security	8	Women
9	Town	Social Security	10	Men

Table 2. The focus groups sessions. Results

	**Results**
**Users' perception**	
**Topics**	
Perception of Quality - Positive Aspects	• Satisfaction with the care provided at primary healthcare centers (PHC).<br>• Good attitude and willingness of the staff.<br>• Users appreciated when PHC offer imaging and laboratory tests, provide medication, and allow consultations with specialist doctors.<br>• Some users noted a difference in the care received at hospitals, where the treatment is not as cordial as in the PHC.<br>• Complaints about lack of respect in PHC and hospitals were infrequent.<br>• Activities promoting physical exercise and healthy habits.

	• Some PHC are highlighted as very comprehensive, offering multiple specialties, and serving as referral centers before sending patients to a hospital.
Barriers to access	• Difficulty in accessing the appointment system.<br>• Excessive waiting times for consultations at PHC and hospitals.<br>• Absenteeism by both users who do not attend their scheduled appointments and professionals who cannot attend due to last-minute obligations.<br>• Limited availability of specialties at PHC.<br>• Difficulty accessing specialty care at higher-complexity levels.<br>• Poorer quality of care, especially in Psychology and Nutrition.<br>• Users complained about the frequent change of medical professionals.<br>• Laboratory services not available at PHC can be obtained at hospitals, with an indication that it is an emergency.
Reaching a diagnosis	• Most users from both healthcare systems became aware of their illness after experiencing symptoms, consulting a general practitioner, and undergoing diagnostic tests.
Access to PHC	• Users go to PHC (Primary Healthcare Centers) for proximity and are referred to second- and third-level hospitals for specialists and complex exams.<br>• Chronic illness patients are treated by family doctors or general practitioners, who refer them to specialists if complications arise.<br>• Family doctors or general practitioners can prescribe psychiatric medication.<br>• Some PHC centers have psychologists, but psychiatric consultations usually require hospital care.<br>• Access to hospital care requires a referral from a primary care physician, a user's appointment request, and a registration process.<br>• Most users report their illness is under control due to routine care at PHC and their adherence to treatment, despite challenges.

Obtaining appointments	• Treatment interruptions due to lack of appointments can occur at any stage of care.<br>• Missed test appointments can lead to referral expiration, forcing users to restart the process.<br>• When unable to secure appointments, users either seek private care or abandon treatment.<br>• Users often visit second-level institutions repeatedly, filing complaints and requesting new orders.<br>• Frustrated by long waits, some users bypass the system and go directly to health centers where they know they can receive care.<br>• For emergencies, users rely on hospital services with shorter wait times.<br>• To avoid delays, expired orders, and medication shortages, users plan appointments ahead, accounting for test result times.
Medication	• When necessary, medication is unavailable and users cannot afford to purchase it, they interrupt treatments based on their own set priorities.<br>• Users reported frequent shortages of supplies and medication.<br>• In cases of medication shortages, staff at PHC inform users about the availability of supplies or any changes in the care schedule, thanks to their relationship with medical and administrative personnel.
Capabilities and Resources	**Financial Situation**<br>• When public system users cannot get specialist appointments in hospitals, they lack the financial resources for private care.<br>• Travel costs and time to reach cities offering services are frequent barriers, causing some users to pay for private care.<br>• Users with financial means would prefer to use the private system.<br>• Economic difficulties prevent users from adhering to a recommended diet, as they cannot afford to prepare separate meals or buy specific foods.<br><br>**Networks**<br>• Users emphasized the importance of building a relationship and trust with professionals, but often find that the professional is no longer at PHC<br>• Users rely on family, friends, and acquaintances to help secure appointments via official channels (phone numbers or apps).<br>• They ask others to call at different times or during hours when they themselves are unavailable, or rely on acquaintances within the system.

Supplement to: Mazzoni A, Roberti J, Guglielmino M, et al. Service delivery redesign for noncommunicable disease management: assessment of needs and solutions through a co-creation process in Argentina. *Glob Health Sci Pract*. 2024;12(6):e2400208. https://doi.org/10.9745/GHSP-D-24-00208

In-depth interviews with stakeholders

We conducted 19 in-depth interviews with health care providers and policymakers from both subsystems, including directors of chronic disease programs, primary care center directors, primary care physicians, psychologists, and secondary care specialists. The interviews assessed several areas: program activities and objectives, available services, population characteristics, interactions with other levels of care, and difficulties, challenges, and facilitators in patient care. All interviews were compiled into a coherent corpus, and the transcripts were uploaded into the qualitative analysis software Atlas.Ti v8.1.3 (Scientific Software Development GmbH, Germany). The coding process followed an abductive approach. *See Table 3 for more details.*

Table 1. Professionals' perception. Results

Professionals' perception.
• They emphasized that they provide good quality care and adequate access to necessary medication.<br>• Problems in the provision of material resources (e.g., computers, certain medical supplies).<br>• The main obstacle: insufficient human resources to meet user demand.<br>• Many professionals have changed their tasks from care to management, leaving certain areas vacant.<br>• Due to the lack of human resources, it is difficult to carry out health promotion or disease prevention activities.<br>• Regarding mental health care, professionals also highlighted difficulties in meeting demand and pointed out that general practitioners often cover this area with the few resources available.<br>• They cannot pursue the training topics proposed by the Ministry due to a lack of time and human resources.<br>• Professionals reported efforts to strengthen and facilitate the referral and counter-referral processes.<br>• They provide training to PHC professionals to address more issues and relieve the system.<br>• Actions to improve communication between health centers.<br>• Lack of counter-referral information from hospitals to primary care centers.<br>• Central Hospital was pointed out as having problems with obtaining appointments.<br>• Professionals assist patients who have difficulty accessing the referral system and use informal networks of contacts in other centers to expedite the referral process.

Supplement to: Mazzoni A, Roberti J, Guglielmino M, et al. Service delivery redesign for noncommunicable disease management: assessment of needs and solutions through a co-creation process in Argentina. *Glob Health Sci Pract*. 2024;12(6):e2400208. https://doi.org/10.9745/GHSP-D-24-00208

People Voice Survey (PVS)

The People's Voice Survey is a survey developed by the QuEST network designed to promote health systems' accountability, track the impact of reforms and policies over time and promote benchmarking across countries and subnational regions. The main results obtained from the province of Mendoza through the PVS are shown below.

QuEST LAC IECS

People's Voice Survey: Argentina Country Brief 2023

QuEST Quality Evidence for Health System Transformation

BACKGROUND

High-quality health systems generate health, trust, and economic benefit while responding to changing population needs. Current measurements of health systems fail to capture people's perspectives at the population level. The People's Voice Survey (PVS) is a new instrument that measures health system performance from the populations' perspective. It assesses use of health care as well as people's experiences, expectations, and confidence in the health system.

METHODS

In September 2022, data were collected from a nationally representative sample of 1,190 adults in Mendoza, Argentina via an online survey administered in Spanish. All data are weighted to represent the population.

Respondent demographics[1]		Overall (N = 1,190)
Age (median) [Min, Max]		47.0 [18.0-93.0]
Female		729 (61.3%)
Urban residency		1,115 (93.9%)
Education (highest level)	Post-secondary	394 (33.2%)
	Secondary	468 (39.4%)
	Primary	297 (25.0%)
Household income (monthly)	Highest (> 130,000 pesos)	251 (21.1%)
	Middle (60,000 - 129,999 pesos)	386 (32.5%)
	Lowest (< 59,999 pesos)	486 (40.8%)
	Unknown	67 (5.6%)
Health insurance	No insurance	0 (0.0%)
	Public insurance	488 (41.0%)
	Social security/military	643 (54.0%)
	Private insurance	59 (5.0%)

1. Denominators of some items may vary slightly from the full sample size due to missingness

KEY FINDINGS

Health care utilization in past 12 months

Average number of health care contacts (all types)	5.7
Average number of in-person facility visits	4.9
Average number of visits for COVID-19	0.4
Average number of virtual or telemedicine contacts	0.4
% of respondents who received mental health care (among those with poor or fair mental health)	37.2%
% of all respondents who had an overnight hospital stay	9.1%
% of all respondents with no unmet need for health care	80.3%

Usual source of care: Percent with usual health care facility or provider's group

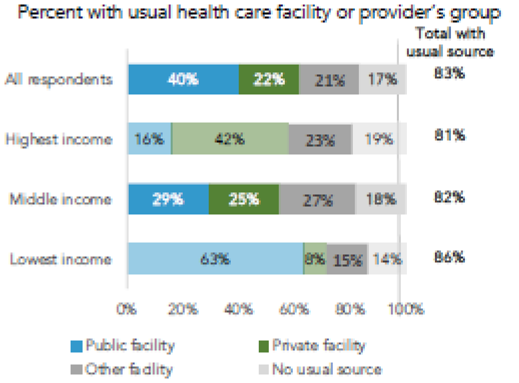

Health system competence

Preventative care services: Percent of eligible population who received service in past 12 months

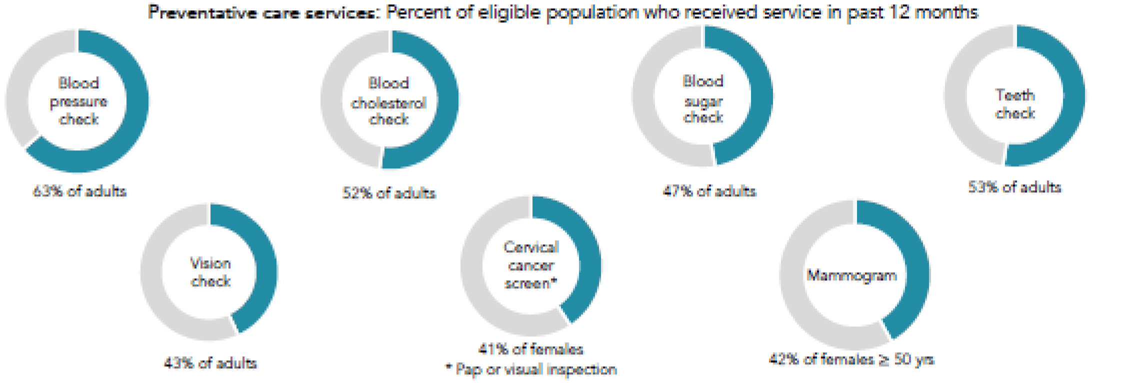

Note: Percentages in this brief may not sum to expected totals due to rounding

Supplement to: Mazzoni A, Roberti J, Guglielmino M, et al. Service delivery redesign for noncommunicable disease management: assessment of needs and solutions through a co-creation process in Argentina. *Glob Health Sci Pract*. 2024;12(6):e2400208. https://doi.org/10.9745/GHSP-D-24-00208

Care competence and user experience

Average[1] quality ratings for last health care visit in past 12 months

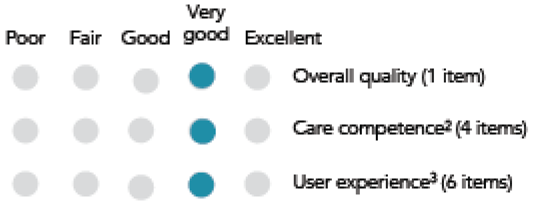

1. Rounded to the closest Likert category
2. Provider skills, knowledge of past visits, explanations, equipment/supplies
3. Respect, courtesy, joint decisions, visit time, wait time, scheduling time

Quality ratings for last health care visit (% of users of facility type in past 12 months)

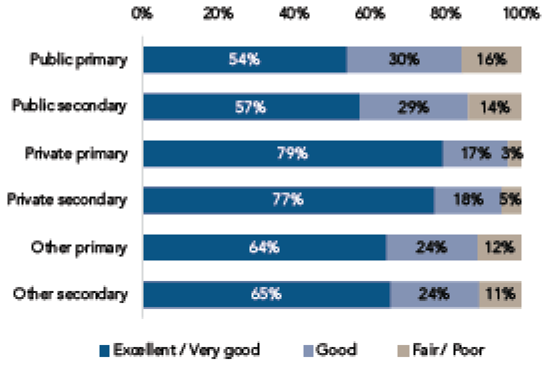

Health system quality

Quality ratings of national public and private health systems (% of all respondents)

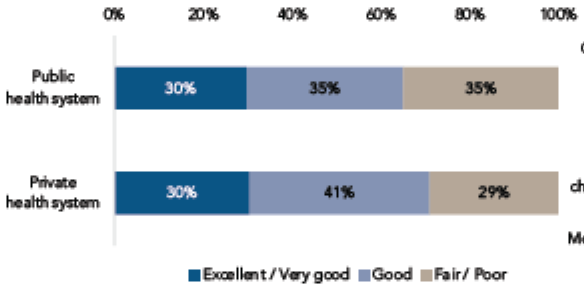

Quality ratings of key primary care services provided by government (% of all respondents)

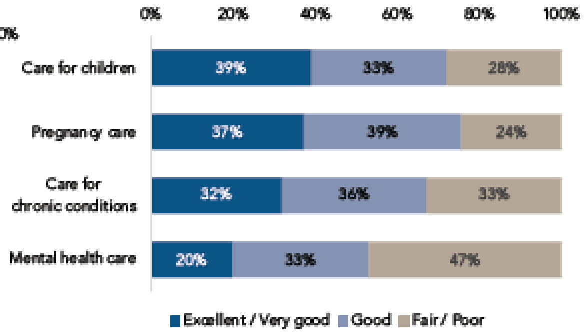

Confidence in and endorsement of the health system

**Confidence in health system as a whole (% of all respondents):** Including public, private, NGO health care facilities/providers		
**Health security:** % very or somewhat confident	Can get good quality care if very sick	65%
	Can afford good quality care if very sick	42%
	Can get and afford good quality care if very sick	33%
**Government considers the public's opinion in health system decisions (% of all respondents):** % very or somewhat confident		27%
**Government's management of the COVID-19 pandemic (% of all respondents):** % excellent or very good		39%

Endorsement: Health system trajectory over past 2 years (% of all respondents)

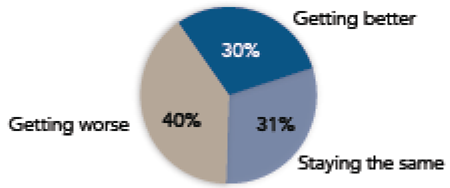

Endorsement: Current health system (% of all respondents)

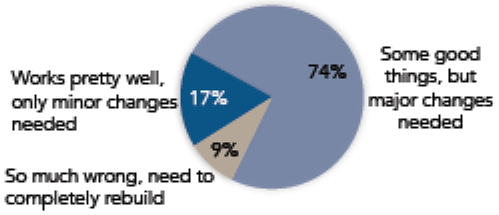

People's Voice Survey: Argentina (Mendoza) Country Brief 2023

Note: Percentages in this brief may not sum to expected totals due to rounding.

Supplement to: Mazzoni A, Roberti J, Guglielmino M, et al. Service delivery redesign for noncommunicable disease management: assessment of needs and solutions through a co-creation process in Argentina. *Glob Health Sci Pract*. 2024;12(6):e2400208. https://doi.org/10.9745/GHSP-D-24-00208

Electronic cohort of patients with diabetes

This was a longitudinal, prospective, real-time survey designed to evaluate the performance of the health care system, assess patients' experiences in managing their disease, and identify gaps along the continuum of care for people with type 2 diabetes. We recruited 252 individuals from the public and social security sectors and followed them virtually (using mobile technology) for 6 months. Cognitive interviews were conducted before recruitment began to test and adjust the instrument. The interviewers were staff from the Ministry of Health in the public sector and staff from the call center in the social security sector, specially trained to conduct the survey and use RedCap for data entry.
A total of 252 patients were studied with 172 individuals from the public sector and 80 from the social security sector. The mean age was 58 years (SD 18.3); 61.3% were female; .41.1% had completed at least secondary education, 19.1% belonged to the lowest household income level, and 53% were employed (Table 4-5).
Regarding the continuity of care, during the follow-up months, a total of 44.4% (57.5% public sector, 16.3% social security) responded to 2 or more telephone surveys. To see more details of the results obtained in this population, refer to the tables and charts below.

Table 1. Patient enrollment

Sector	Screening	Date the last screening	Continued participation (%)	Eligible (%)	Enrolled (%)	Baseline form (%)	Under follow-up (%)	Completed (%)
OSEP	145	2023-07-11	85 (58.62)	84 (98.82)	81 (96.43)	79 (94.04)	4 (5)	4 (5)
Public	268	2023-08-29	255 (95.15)	175 (68.63)	174 (99.43)	174 (99.43)	97 (55.7)	90 (51.7)
Total	413		340 (82.32)	259 (76.18)	255 (98.46)	253 (97.7)	101 (39.9)	94 (36.8)

Table 2. Sociodemographic characteristics

Characteristic	Total, N = 252 (%)
**Mean age**	58 years (SD 18.3)
**Gender**<br>Female	61.3 %

Supplement to: Mazzoni A, Roberti J, Guglielmino M, et al. Service delivery redesign for noncommunicable disease management: assessment of needs and solutions through a co-creation process in Argentina. *Glob Health Sci Pract*. 2024;12(6):e2400208. https://doi.org/10.9745/GHSP-D-24-00208

**Education**<br>Primary or inferior<br>Secondary or superior	 58 %<br>42 %
**Work or any other source of income**	53.2%
**Income***<br>Low (< 59,999)<br>Medium (60,000 - 129,999 pesos)<br>High (> 130,000 pesos)<br>No answer	 46.2%<br>30.3%<br>6.1%<br>17.4%
**Health insurance**<br>Public<br>OSEP<br>No answer	 68.1%<br>31.5%<br>0.4%
**Self-reported health status as *'Excellent' or 'Very good'***	10.6%
**Positive activation**	72.4%

*Only reported for those with an income

Table 3. General health characteristics

Characteristic	Total, N = 252 (%)
**Diabetes or high blood sugar (mentioned by a HP)**<br>Yes<br>No<br>No answer	 <br>97.2%<br>2.4%<br>0.4%
**Years since diagnosis****<br>Ten years or less<br>More than 10 years	 68.8%<br>31.3%
**Treatment with oral hypoglycemic drugs****	80.4%
**Treatment with Insulin****	44.6%
**IMC ≥ 30**	59.6%
**Smoking**	23.7%
**Alcohol**	45.7%
**Salt in meals**	69.4%
**Ultra-processed foods in the diet**	86.9%
**Hypertension***	75.5%
**Hypercholesterolemia****	64.5%

Supplement to: Mazzoni A, Roberti J, Guglielmino M, et al. Service delivery redesign for noncommunicable disease management: assessment of needs and solutions through a co-creation process in Argentina. *Glob Health Sci Pract*. 2024;12(6):e2400208. https://doi.org/10.9745/GHSP-D-24-00208

**Myocardial infarction or stroke***	22.4%
**Chronic kidney disease***	16.7%

**Only reported for those with DBT

Figure 1. Reasons why medical visit was not done (Total N= 124)

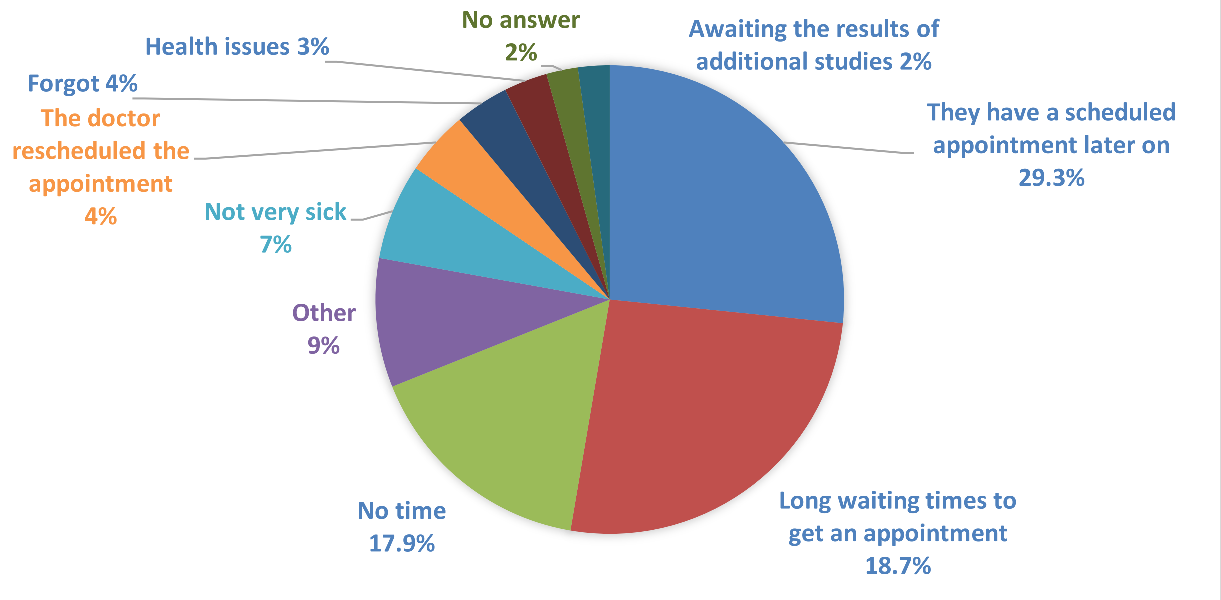

Figure 2. Out-of-pocket cost (Total N=94)

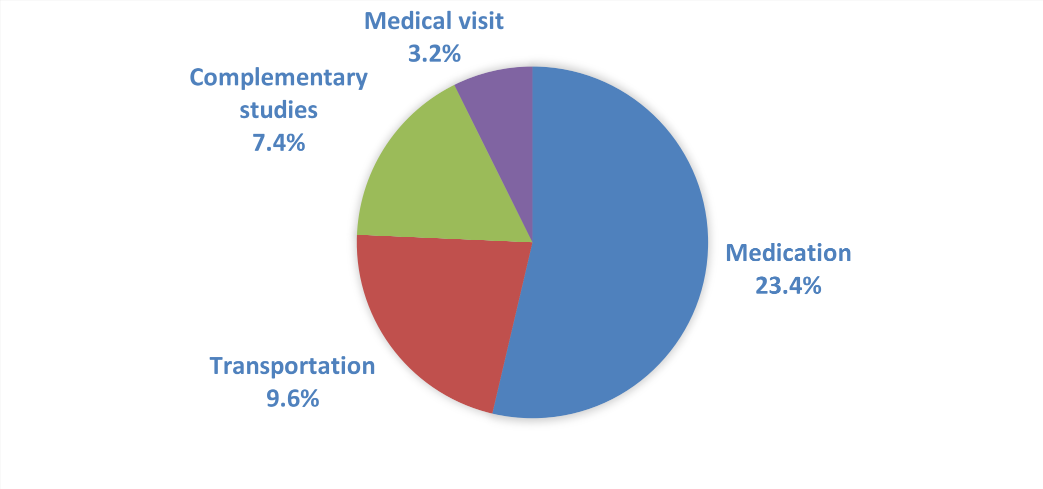

Table 4. Positive user experience.

Positive user experience	Total (N = 101)
Respect	93.6%
Communication	91.5%
Autonomy	83.9%

Non-discrimination	97.9%
Self-reported quality of healthcare received for their diabetes problem as *'Excellent' or 'Very good'*	63.3%

Knowledge test to healthcare providers

This assessment was embedded in a training course offered by the Ministry of Health to public and social security health providers in Mendoza. The skills assessment questions were based on current provincial and national guidelines. Our aim was to determine whether there were any weaknesses in providers' ability to meet the needs of patients with chronic conditions. Participation was voluntary and 99 out of 300 invited health care providers completed the survey. *See table 6 for more details.*

Table 1. Knowledge test to healthcare providers. Results.

Questions	% Correct Answers N=99
Percentage of controlled hypertensive patients in Argentina	34%
Correct sphygmomanometer for blood pressure measurement	57%
Target range for blood pressure control	62%
Optimal frequency for blood pressure control	48%
Cardinal symptoms of depression	99%
Description of depression symptoms	92%
Differential diagnosis of depression	99%
Best option for depression treatment	68%
Case 1 for type 2 diabetes treatment	52%
Case 2 for type 2 diabetes treatment	45%
Case 3 for type 2 diabetes treatment	32%
Diagnosis of type 2 diabetes	83%
Lifestyle changes for type 2 diabetes	96%

Consensus process to develop recommendations

The purpose of the consensus process was to reach agreement on recommendations and generate a comprehensive list of feasible and culturally appropriate interventions specifically adapted to the

Mendoza health system. The aim was to provide the province with evidence-based recommendations that would have a positive impact on the primary care network, particularly in chronic disease management.

A modified RAND UCLA Delphi method was used to reach consensus. This method is widely used to reach consensus among groups of experts or stakeholders on a particular issue. The Delphi process is structured and iterative to gather opinions and ideas from a group of participants. It is particularly valuable in situations where face-to-face meetings are not feasible, or where the influence of dominant individuals or group dynamics needs to be minimized. The method uses iterative rounds of feedback and revision, allowing participants to re-evaluate their positions in light of the collective input received. This iterative process promotes convergence towards consensus, leading to a final set of recommendations or decisions. In addition, the modified method incorporates elements of the RAND/UCLA Adequacy Method, which adds a structured approach to scoring and evaluating panelists' responses.

Twenty-two professionals participated in the first two rounds of voting to evaluate the recommendations according to five criteria (potential impact, required resources, feasibility, acceptability and measurement) proposed by GRADE from the Evidence to Decision. *See table 8.* The final round, attended by 20 participants, was held in Mendoza to discuss the recommendations that showed disagreements in the scoring of the evaluation criteria, and to reach a consensus whenever possible. The findings of the consensus showed numerous actions that are supported by evidence and expert agreement for implementation, which will guide future changes in the system or be part of the research agenda. The initiatives with the highest scores were seven. *See Table 9.*

Table 1. Definition criteria proposed by GRADE.

Criteria	Definition
**Potential impact**	What is the magnitude of the potential impact of this recommendation or practice for improving the quality of care at the primary level?
**Required resources**	What is the size of the resource requirements (money, time, human resources, etc.) needed to implement this recommendation?
**Feasibility**	Will the recommendation require extraordinary additional resources or those beyond the reach of the public health system?
**Acceptability**	Do you think the recipients of the recommendation will accept this intervention? Is it acceptable to those involved (patients, professionals, other actors within the health system)?
**Measurement**	Is there an indicator that can measure whether the recommendation is being implemented without additional resources or with very few additional resources?

Table 2. List of the final recommendations developed through the Delphi consensus.

Recommendations
1. Implement the digital transformation law.
2. Promote mechanisms for user participation in the design and provision of healthcare in the health system.
3. Develop skills in users to make them active participants in healthcare.
4. Utilize information and communication technologies to provide information on specific conditions, clinical reminders, screenings, and appointment cancellations.

5. Develop co-produced definitions of quality care involving both users and healthcare personnel at the primary care level
6. Implement an incident reporting system in primary care centers.
7. Implement performance metrics to assess professional practice
